# Supplementary material for: Immiscible hydrous Fe–Ca–P melt and the origin of iron oxide-apatite ore deposits
Source: Nat Commun. 2018 Apr 12;9:1415. doi: 10.1038/s41467-018-03761-4 (PMC5897329; doi:10.1038/s41467-018-03761-4)
Supplement: Supplementary file 1 — Supplementary Information(PDF 270 kb) [file 41467_2018_3761_MOESM1_ESM.pdf]

Supplementary information

Immiscible hydrous Fe-Ca-P melt and the origin of iron oxide-apatite ore deposits

Hou et al.

Supplementary Table 1. End-member compositions used to prepare the starting experimental powders. Fayalite and magnetite compositions are from ref. [1], M1 and M2 are mixtures of fayalite and magnetite in proportions 30:70 and 60:40, respectively. The rhyolite composition is from ref. [2].

	SiO_2	TiO_2	Al_2O_3	FeOtot	MnO	MgO	CaO	Na_2O	K_2O	P_2O_5	Total
Fayalite	29.25	0.02	0.07	68.27	1.85	0.45	0.1				100
Magnetite	0.83	14.64	0.22	83.74	0.37	0.16	0.05				100
M1	9.36	10.25	0.17	79.1	0.82	0.25	0.06				100
M2	17.88	5.86	0.13	74.46	1.26	0.33	0.08				100
Rhyolite	74.54	0.3	11.78	4.21	0.09	0.76	0.8	2.41	5.06	0.04	100

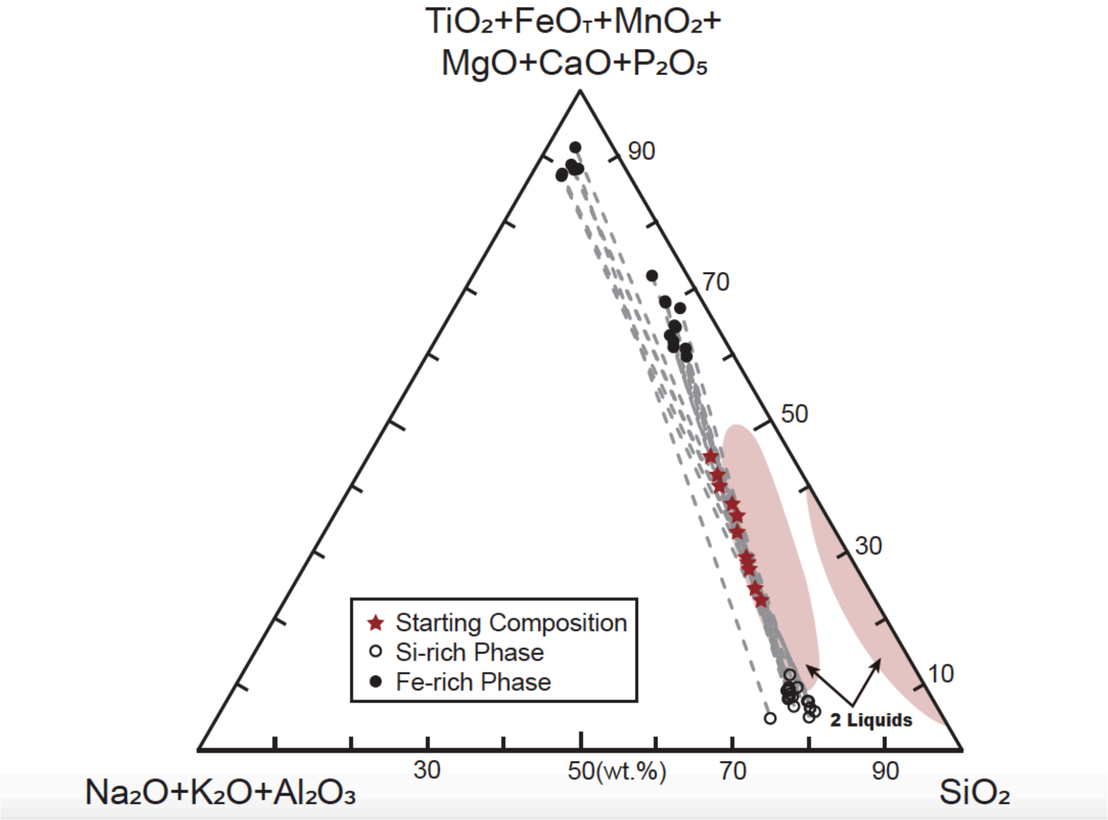

Supplementary Figure 1 Compositional field for starting compositions. Data are plotted onto the ternary diagram SiO_2 – Na_2O+K_2O+Al_2O_3 – TiO_2+FeO+MnO+MgO+CaO+P_2O_5 and compared with the two-liquid immiscibility fields in the Leucite–Fayalite–Quartz system investigated[3]. The immiscible pairs produced in this study are also plotted for comparison.

Supplementary Table 2. Compositions (wt.%) of starting materials. P1 = 58.5% rhyolite + 39% M2 + 0.5% CaF_2, P2 = 58.5% rhyolite + 39% M1 + 0.5% CaF_2, and P3 = 79.6% rhyolite + 19.9% M2 + 0.5% CaF_2

Sample	Proportions (wt.%)	SiO_2	TiO_2	Al_2O_3	FeOtot	MnO	MgO	CaO	Na_2O	K_2O	P_2O_5	F	S	H_2O	Total
LP02	P1 + 1.09% $Ca_3(PO_4)_2$ + 2% H_2O	48.93	2.38	6.71	30.47	0.53	0.55	2.26	1.36	2.86	0.50	0.47		2.00	100.06
LP03	P1 + 1.09% $Ca_3(PO_4)_2$ + 4% H_2O	47.93	2.33	6.58	29.85	0.52	0.54	2.25	1.34	2.80	0.50	0.46		4.00	100.12
LP04	P1 + 3.28% $Ca_3(PO_4)_2$ + 2% H_2O	48.89	2.38	6.71	30.45	0.53	0.55	2.26	1.36	2.86	1.52	0.47		2.00	99.99
LP05	P1 + 3.28% $Ca_3(PO_4)_2$ + 4% H_2O	47.86	2.33	6.57	29.81	0.51	0.54	2.25	1.33	2.80	1.52	0.46		4.00	99.99
LP06	P1 + 3.28% $Ca_3(PO_4)_2$ + 6% H_2O	46.83	2.28	6.43	29.17	0.50	0.53	2.24	1.31	2.74	1.52	0.45		6.00	99.99
HP01; HP07; HP16	P3 + 2.5% $Ca_3(PO_4)_2$	61.60	1.38	9.21	17.79	0.32	0.66	2.00	1.88	3.94	1.18	0.49			100.43
HP02; HP08; HP17	P3 + 5% $Ca_3(PO_4)_2$	60.02	1.34	8.97	17.34	0.31	0.64	3.33	1.83	3.84	2.32	0.48			100.42
HP03; HP09; HP18	P3 + 10% $Ca_3(PO_4)_2$	56.86	1.27	8.50	16.43	0.29	0.61	6.01	1.73	3.64	4.60	0.45			100.40
HP04; HP10	P3 + 2.5% $Ca_3(PO_4)_2$ + 6% H_2O	57.90	1.29	8.66	16.73	0.30	0.62	1.88	1.77	3.71	1.10	0.46		6.00	100.41
HP05; HP11	P3 + 5% $Ca_3(PO_4)_2$ + 6% H_2O	56.42	1.26	8.43	16.30	0.29	0.60	3.13	1.72	3.61	2.18	0.45		6.00	100.39
HP06; HP12	P3 + 10% $Ca_3(PO_4)_2$ + 6% H_2O	53.45	1.19	7.99	15.44	0.27	0.57	5.65	1.63	3.42	4.33	0.42		6.00	100.37
HP13	P2 + 2.5% $Ca_3(PO_4)_2$	47.02	4.15	6.93	33.15	0.37	0.54	1.84	1.41	2.95	1.17	0.49			100.02
HP14	P2 + 5% $Ca_3(PO_4)_2$	45.82	4.05	6.75	32.30	0.36	0.53	3.18	1.37	2.87	2.32	0.48			100.02
HP15	P2 + 10% $Ca_3(PO_4)_2$	43.40	3.83	6.39	30.60	0.34	0.50	5.87	1.30	2.72	4.60	0.45			100.02
HP22; HP25	P3 + 2.5% $Ca_3(PO_4)_2$ + 8% FeS	56.67	1.27	8.47	22.78	0.29	0.60	1.84	1.73	3.63	1.08	0.45	3.02		101.82
HP23; HP26	P3 + 5% $Ca_3(PO_4)_2$ + 8% FeS	55.22	1.23	8.26	22.36	0.28	0.59	3.07	1.68	3.54	2.13	0.44	3.02		101.81
HP24; HP27	P3 + 10% $Ca_3(PO_4)_2$ + 8% FeS	52.31	1.17	7.82	21.52	0.27	0.56	5.53	1.60	3.35	4.24	0.41	3.02		101.79

Supplementary Table 3. Experimental conditions and phase assemblages

Sample	T [°C]	P [MPa]	Run duration (days)	fO_2	aH_2O	Phases
LP02	1010	100	2	FMQ+3.2	0.85	2-liq, Ap, Mt, Ti-Hem
LP03	1010	100	2	FMQ+3.3	1.00	2-liq, Ap, Mt, Ti-Hem
LP04	1010	100	3	FMQ+3.1	0.70	2-liq, Mt, Ti-Hem
LP05	1010	100	3	FMQ+3.2	0.82	2-liq, Mt, Ti-Hem, Tri
LP06	1010	100	3	FMQ+3.3	1.00	2-liq, Mt, Ti-Hem
HP01	1000	100	7	FMQ+0.5	0.1**	Liq, Mt, Ap, Fa, Tri
HP02	1000	100	7	FMQ+0.5	0.1**	Liq, Mt, Ap, Fa, Tri
HP03	1000	100	7	FMQ+0.5	0.1**	2-liq, Mt, Ap, Fa, Tri
HP04	1000	100	7	FMQ+0.5	0.56	Liq, Cpx, Fa, Mt, Ap
HP05	1000	100	7	FMQ+0.5	1.00	2-liq*, Mt, Ap, Tri
HP06	1000	100	7	FMQ+0.5	0.61	2-liq*, Mt, Ilm, Ap, Fa, Tri
HP07	1020	100	7	FMQ+0.5	0.1**	2-liq, Mt, Ap, Fa, Tri
HP08	1020	100	7	FMQ+0.5	0.1**	2-liq, Mt, Ap, Fa
HP09	1020	100	7	FMQ+0.5	0.1**	2-liq, Mt, Ap, Fa
HP10	1020	100	5	FMQ+0.5	0.60	Liq, Mt, Ap
HP11	1020	100	5	FMQ+0.5	0.69	2-liq*, Mt, Ap
HP12	1020	100	5	FMQ+0.5	0.69	2-liq, Mt, Ap, Fa
HP13	1000	100	7	FMQ+0.5	0.1**	2-liq*, Mt, Ap, Fa, Tri
HP14	1000	100	7	FMQ+0.5	0.1**	2-liq*, Mt, Ap, Fa, Tri
HP15	1000	100	7	FMQ+0.5	0.1**	2-liq*, Mt, Ap, Fa, Tri
HP16	1040	100	3	FMQ+0.5	0.1**	Liq, Mt, Ap, Tri
HP17	1040	100	3	FMQ+0.5	0.1**	Liq, Mt, Ap, Tri
HP18	1040	100	3	FMQ+0.5	0.1**	Liq, Mt, Ap, Tri
HP22	1020	100	5	FMQ+0.5	0.1**	2-liq, Mt, Ap, Fa, Tri, Sul
HP23	1020	100	5	FMQ+0.5	0.1**	2-liq, Mt, Ap, Fa, Tri, Sul
HP24	1020	100	5	FMQ+0.5	0.1**	2-liq, Mt, Ap, Fa, Tri, Sul
HP25	1000	100	7	FMQ+0.5	0.1**	2-liq, Mt, Ap, Fa, Tri, Sul
HP26	1000	100	7	FMQ+0.5	0.1**	2-liq, Mt, Ap, Fa, Tri, Sul
HP27	1000	100	7	FMQ+0.5	0.1**	2-liq, Mt, Ap, Fa, Tri, Sul

Abbreviations: Ap –apatite, Mt – magnetite, Ilm – ilmenite, Ti-Hem – Ti-rich hematite (Ilm-Hem solid solution), Fa – fayalite, Tri – tridymite, Cpx – clinopyroxene, Sul – sulfide, liq – liquid(s).

* Fe-rich globules too small to be measured; ** Activity of H_2O is estimated as nominally dry.

References

1. Borrok, D. M., Kelser S. E., Boer R. H. & Essene E. J. The Vergenoeg magnetite-fluorite deposit, South Africa; support for a hydrothermal model for massive iron oxide deposits. *Econ. Geol.* **93**, 564–586 (1998).
2. Hou, T., et al. Experimental study of liquid immiscibility in the Kiruna-type Vergenoeg iron–fluorine deposit, South Africa. *Geochim. Cosmochim. Acta*, **203**, 303–322 (2017).
3. Roedder, E. Low temperature liquid immiscibility in the system K_2O-FeO-Al_2O_3-SiO_2. *Am. Miner*. **36**, 282–286 (1951).
