# Supplementary material for: Immiscible hydrous Fe–Ca–P melt and the origin of iron oxide-apatite ore deposits
Source: Nat Commun. 2018 Apr 12;9:1415. doi: 10.1038/s41467-018-03761-4 (PMC5897329; doi:10.1038/s41467-018-03761-4)
Supplement: Supplementary file 3 — Description of Additional Supplementary Files(PDF 165 kb) [file 41467_2018_3761_MOESM3_ESM.pdf]

Description of Additional Supplementary Files

File Name: Supplementary Data 1
Description: Electron microprobe analyses of run products
