# Supplementary material for: Immiscible hydrous Fe–Ca–P melt and the origin of iron oxide-apatite ore deposits
Source: Nat Commun. 2018 Apr 12;9:1415. doi: 10.1038/s41467-018-03761-4 (PMC5897329; doi:10.1038/s41467-018-03761-4)
Supplement: Supplementary file 4 — Supplementary Data 1(DOCX 131 kb) [file 41467_2018_3761_MOESM4_ESM.docx]

**Supplementary Data 1** Electron microprobe analyses of run products

| **Sample** | **Phases** | **SiO_2_** | **TiO_2_** | **Al_2_O_3_** | **FeO_tot_** | **MnO** | **MgO** | **CaO** | **Na_2_O** | **K_2_O** | **P_2_O_5_** | **F** | **SO_3_** | **Total** | **H_2_O** |
| --- | --- | --- | --- | --- | --- | --- | --- | --- | --- | --- | --- | --- | --- | --- | --- |
| **LP02** | **gl Si(10)^1^** | 74.13(0.96)^2^ | 0.68(0.06) | 9.98(0.38) | 2.99(0.28) | 0.15(0.02) | 0.25(0.02) | 0.52(0.07) | 2.20(0.14) | 4.54(0.12) | 0.38(0.02) | 0.47(0.02) |  | 95.99 | 3.07(0.13) |
|  | **gl Fe(10)** | 3.40(0.09) | 0.98(0.04) | 5.72(0.25) | 19.15(0.31) | 3.53(0.15) | 7.23(0.06) | 27.20(0.30) | 2.19(0.03) | 0.65(0.00) | 26.43(0.09) | 2.71(0.02) |  | 96.48 | 1.66(0.27) |
|  | **Ti-Hem(2)** | 0.22(0.02) | 19.41(0.03) | 0.55(0.08) | 71.37(0.28) | 0.35(0.15) | 0.66(0.00) |  |  |  |  |  |  | 92.56 |  |
|  | **Mt(3)** | 0.12(0.01) | 3.31(0.04) | 1.52(0.01) | 85.62(0.55) | 1.03(0.07) | 1.19(0.15) |  |  |  | - | - |  | 92.85 |  |
| **LP03** | **gl Si(10)** | 73.23(0.59) | 0.73(0.01) | 9.42(0.17) | 3.05(0.14) | 0.21(0.02) | 0.33(0.01) | 0.74(0.04) | 1.69(0.20) | 4.21(0.09) | 0.62(0.07) | 0.55(0.03) |  | 94.39 | 3.89(0.42) |
|  | **gl Fe(10)** | 3.33(0.05) | 0.98(0.02) | 5.19(0.08) | 18.88(0.49) | 5.66(0.16) | 7.48(0.52) | 27.16(0.08) | 2.5(0.11) | 0.69(0.02) | 25.71(0.61) | 2.51 (0.01) |  | 96.98 | 1.94 |
|  | **Ti-Hem(2)** | 0.37(0.02) | 19.36(0.25) | 0.57(0.09) | 71.03(1.04) | 0.35(0.20) | 0.62(0.00) |  |  |  |  |  |  | 92.30 |  |
|  | **Mt(4)** | 0.07(0.04) | 3.37(0.12) | 1.49(0.09) | 86.85(0.36) | 0.94(0.13) | 1.06(0.09) |  |  |  |  |  |  | 93.78 |  |
| **LP04** | **gl Si(5)** | 70.97(0.29) | 0.57(0.04) | 9.28(0.52) | 3.20(0.27) | 0.12(0.03) | 0.16(0.03) | 0.15(0.03) | 1.86(0.07) | 4.23(0.02) | 2.93(0.03) | 0.33(0.01) |  | 97.70 | 2.66(0.24) |
|  | **gl Fe(5)** | 3.86(1.13) | 0.99(0.13) | 3.85(0.37) | 34.15(0.73) | 3.06(0.10) | 4.87(0.21) | 5.13(0.10) | 1.81(0.09) | 0.97(0.04) | 39.40(1.13) | 0.51(0.03) |  | 98.61 | 1.03(0.09) |
|  | **Ti-Hem(11)** | 0.02(0.01) | 16.66(0.10) | 0.56(0.09) | 77.46(0.34) | 0.19(0.02) | 0.14(0.01) |  |  |  |  |  |  | 95.03 |  |
|  | **Mt(12)** | 0.09(0.03) | 2.37(0.04) | 1.41(0.03) | 91.39(0.34) | 0.48(0.11) | 0.31(0.02) |  |  |  |  |  |  | 96.06 |  |
| **LP05** | **gl Si(5)** | 73.71(0.16) | 0.60(0.03) | 9.69(0.06) | 2.48(0.36) | 0.06(0.06) | 0.12(0.04) | 0.21(0.02) | 1.47(0.08) | 4.77(0.14) | 2.84(0.48) | 0.69(0.01) |  | 95.82 | 3.07(0.13) |
|  | **gl Fe(5)** | 3.09(1.05) | 1.53(0.20) | 2.18(0.23) | 36.36(0.36) | 2.99(0.15) | 5.04(0.16) | 5.74(0.31) | 1.90(0.08) | 0.77(0.11) | 39.37(0.55) | 0.60(0.03) |  | 99.57 | 1.63(0.26) |
|  | **Ti-Hem(11)** | 0.05(0.01) | 24.64(0.12) | 0.74(0.02) | 71.65(0.03) | 0.24(0.03) | 0.25(0.08) |  |  |  |  |  |  | 97.57 |  |
|  | **Mt(8)** | 0.10(0.02) | 5.54(0.06) | 2.20(0.10) | 87.45(0.26) | 0.53(0.11) | 0.40(0.11) |  |  |  |  |  |  | 96.22 |  |
| **LP06** | **gl Si(5)** | 71.39(0.39) | 0.54(0.03) | 9.22(0.42) | 3.19(0.22) | 0.11(0.03) | 0.16 (0.03) | 0.16(0.02) | 1.84(0.08) | 4.23(0.08) | 2.96(0.07) | 0.31(0.01) |  | 97.70 | 3.80(0.20) |
|  | **gl Fe(5)** | 4.55(0.82) | 1.02(0.10) | 3.90(0.15) | 32.63(0.31) | 3.28(0.16) | 5.22(0.22) | 5.53(0.14) | 1.74(0.09) | 1.00(0.04) | 39.02(0.37) | 0.48(0.03) |  | 98.37 | 1.54(0.07) |
|  | **Ti-Hem(3)** | 0.03(0.04) | 14.84(0.08) | 0.67(0.00) | 78.60(0.02) | 0.14(0.06) | 0.14(0.03) |  |  |  |  |  |  | 94.42 |  |
|  | **Mt(4)** | 0.10(0.05) | 2.00(0.09) | 1.46(0.10) | 90.71(0.10) | 0.59(0.13) | 0.34(0.01) |  |  |  |  |  |  | 95.20 |  |
| **HP01** | **gl(20)** | 72.30(0.80) | 0.27(0.03) | 11.13(0.49) | 5.13(0.36) | 0.17(0.08) | 0.30(0.03) | 0.96(0.08) | 2.32(0.11) | 4.81(0.10) | 0.38(0.12) | 0.13(0.01) |  | 97.83 | 0.19(0.03) |
|  | **Mt(3)** | 0.97(0.55) | 11.20(0.18) | 2.48(0.33) | 78.39(0.16) | 0.57(0.03) | 0.66(0.00) |  |  |  |  |  |  | 94.42 |  |
|  | **Fa(2)** | 28.84(0.08) | 0.37(0.08) |  | 62.38(0.15) | 2.61(0.01) | 4.78(0.07) | 0.30(0.09) |  |  |  |  |  | 99.28 |  |
| **HP02** | **gl(20)** | 74.16(0.76) | 0.24(0.02) | 11.80(1.37) | 3.13(0.19) | 0.15(0.07) | 0.42(0.06) | 0.96(0.07) | 2.33(0.15) | 4.89(0.07) | 0.37(0.08) | 0.09(0.01) |  | 98.50 |  |
|  | **Mt(1)** | 0.20 | 6.30 | 2.24 | 83.89 | 0.79 | 1.22 |  |  |  |  |  |  | 94.65 |  |
|  | **Fa(1)** | 30.36 | 0.10 | 0.03 | 60.91 | 2.22 | 5.75 | 0.24 |  |  |  |  |  | 99.61 |  |
| **HP03** | **gl Si(13)** | 72.94(1.38) | 0.36(0.11) | 11.26(0.37) | 4.53(1.12) | 0.16(0.07) | 0.27(0.04) | 1.16(0.17) | 2.17(0.23) | 4.78(0.21) | 0.46(0.25) | 0.12(0.01) |  | 98.15 |  |
|  | **gl Fe(5)** | 31.26(0.69) | 2.13(0.07) | 6.14(0.92) | 35.53(1.19) | 1.05(0.14) | 2.17(0.13) | 8.49(0.16) | 0.60(0.03) | 0.40(0.01) | 11.03(0.52) | n.d. |  | 98.97 |  |
|  | **Mt(4)** | 0.33(0.46) | 6.97(0.95) | 1.40(0.57) | 83.46(1.03) | 0.60(0.11) | 0.73(0.10) |  |  |  |  |  |  | 93.55 |  |
|  | **Fa(1)** | 62.27 | 0.32 |  | 62.27 | 2.60 | 4.73 | 0.37 | 0.01 |  |  |  |  | 99.20 |  |
| **HP04** | **gl(6)** | 71.07(0.66) | 0.25(0.03) | 10.16(0.20) | 6.45(0.13) | 0.24(0.06) | 0.27(0.03) | 0.95(0.02) | 2.21(0.06) | 4.54(0.08) | 0.63(0.04) | 0.15(0.01) |  | 96.93 | 2.31(0.06) |
|  | **Cpx(4)** | 44.47(0.65) | 0.56(0.01) | 6.55(0.14) | 18.18(0.55) | 0.93(0.22) | 12.55(0.35) | 10.69(0.41) | 1.92(0.04) | 1.20(0.05) |  |  |  | 97.06 |  |
|  | **Mt(4)** | 0.38(0.09) | 10.02 (0.22) | 2.57 (0.10) | 81.49(0.55) | 0.58(0.05) | 0.34(0.04) |  |  |  |  |  |  | 94.38 |  |
|  | **Fa(5)** | 30.49(0.34) | 0.14(0.01) | 0.21(0.11) | 61.34(0.56) | 2.13(0.11) | 5.54(0.51) | 0.19(0.05) |  |  |  |  |  | 100.04 |  |
| **HP05** | **gl Si(6)** | 67.01(1.12) | 0.51(0.03) | 9.85(0.36) | 7.52(0.57) | 0.25(0.07) | 0.57(0.07) | 1.37(0.10) | 1.92(0.10) | 4.20(0.12) | 1.00(0.06) | 0.14(0.01) |  | 94.36 | 3.84(0.16) |
|  | **Mt(5)** | 0.25(0.07) | 7.03(0.10) | 1.65(0.48) | 85.13(0.43) | 0.61(0.07) | 0.69(0.03) | 0.03(0.02) |  |  |  |  |  | 95.39 |  |
| **HP06** | **gl Si(6)** | 71.33(0.59) | 0.52(0.04) | 11.39(0.19) | 3.73(0.17) | 0.05(0.05) | 0.14(0.03) | 1.07(0.04) | 2.41(0.07) | 5.16(0.08) | 0.30(0.10) | 0.10(0.01) |  | 96.20 | 2.56(0.08) |
|  | **Mt(5)** | 0.10(0.12) | 2.48(0.28) | 1.14(0.25) | 84.66(1.62) | 1.21(0.21) | 1.22(0.30) | 0.08(0.02) |  |  |  |  |  | 90.89 |  |
|  | **Ilm(5)** | 0.07(0.02) | 53.50(0.19) | 0.23(0.10) | 46.83(0.37) | 0.69(0.05) | 0.16(0.01) | 0.03(0.02) |  |  |  |  |  | 101.63 |  |
|  | **Fa(4)** | 29.36(0.53) | 0.54(0.10) | 0.47(0.51) | 66.73(1.34) | 1.40(0.07) | 1.18(0.04) | 0.70(0.50) |  |  |  |  |  | 99.49 |  |
| **HP07** | **gl Si(6)** | 71.06(0.47) | 0.56(0.03) | 10.94(0.47) | 6.26(0.31) | 0.16(0.03) | 0.28(0.02) | 1.12(0.06) | 2.31(0.06) | 4.73(0.06) | 0.67(0.09) | 0.15(0.01) |  | 98.23 |  |
|  | **gl Fe(6)** | 29.98(2.09) | 2.49(0.09) | 5.16(0.39) | 34.65(0.86) | 1.16(0.13) | 2.00(0.21) | 7.25(0.64) | 0.78(0.09) | 0.56(0.04) | 11.97(1.31) | 0.33(0.05) |  | 96.34 |  |
|  | **Mt(5)** | 0.23(0.04) | 20.66(0.18) | 2.72(0.10) | 74.05(0.27) | 0.59(0.05) | 0.28(0.02) | 0.03(0.02) |  |  |  |  |  | 98.55 |  |
|  | **Fa(5)** | 29.94(0.26) | 0.19(0.09) | 0.06(0.03) | 66.11(0.43) | 1.63(0.09) | 2.88(0.25) | 0.21(0.01) |  |  |  |  |  | 101.03 |  |
| **HP08** | **gl Si(6)** | 71.18(0.79) | 0.28(0.01) | 10.14(0.22) | 6.46(0.12) | 0.27(0.06) | 0.53(0.03) | 1.13(0.03) | 1.73(0.66) | 4.31(0.20) | 0.75(0.04) | 0.12(0.01) |  | 96.90 |  |
|  | **gl Fe(6)** | 26.19(1.55) | 1.34(0.45) | 3.71(0.26) | 35.60(2.03) | 1.91(0.08) | 4.21(0.14) | 7.86(0.63) | 0.58(0.10) | 0.39(0.07) | 14.88(0.69) | 0.31(0.09) |  | 96.97 |  |
|  | **Mt(5)** | 0.35(0.02) | 7.98(0.11) | 2.20(0.08) | 83.89(0.73) | 0.68(0.05) | 0.72(0.04) | 0.09(0.06) |  |  |  |  |  | 95.92 |  |
| **HP09** | **gl Si(6)** | 70.26(0.37) | 0.47(0.02) | 10.44(0.17) | 6.48(0.26) | 0.16(0.05) | 0.29(0.03) | 1.40(0.06) | 2.20(0.08) | 4.65(0.08) | 0.65(0.07) | 0.12(0.02) |  | 97.11 |  |
|  | **gl Fe(6)** | 32.66(0.69) | 1.90(0.12) | 4.53(0.26) | 33.85(0.94) | 1.04(0.06) | 1.93(0.13) | 8.11(0.08) | 0.83(0.07) | 0.59(0.02) | 10.35(0.40) | 0.27(0.01) |  | 96.05 |  |
|  | **Mt(5)** | 0.33(0.07) | 17.77(1.11) | 2.15(0.03) | 76.52(0.99) | 0.54(0.10) | 0.45(0.05) | 0.09(0.04) |  |  |  |  |  | 97.84 |  |
|  | **Fa(5)** | 30.07(0.42) | 0.19(0.05) | 0.08(0.06) | 63.66(0.48) | 1.33(0.08) | 4.91(0.07) | 0.28(0.05) |  |  |  |  |  | 100.55 |  |
| **HP10** | **gl(5)** | 66.43(0.61) | 0.35(0.01) | 9.90(0.13) | 7.70(0.15) | 0.35(0.04) | 0.62(0.04) | 1.19(0.09) | 2.08(0.08) | 4.15(0.09) | 0.89(0.05) | 0.17(0.01) |  | 93.83 | 2.37(0.09) |
|  | **Mt(10)** | 0.26(0.14) | 7.84(0.23) | 1.84(0.15) | 83.42(1.01) | 0.67(0.06) | 0.66(0.03) | 0.06(0.03) |  |  |  |  |  | 94.74 |  |
| **HP11** | **gl Si(4)** | 66.55(0.47) | 0.41(0.02) | 10.02(0.47) | 7.74(0.11) | 0.27(0.03) | 0.62(0.06) | 1.35(0.04) | 2.15(0.09) | 4.25(0.05) | 0.95(0.09) | 0.13(0.00) |  | 94.44 | 2.10(0.08) |
|  | **Mt(12)** | 0.27(0.12) | 9.81(0.49) | 1.87(0.08) | 81.52(0.86) | 0.54(0.06) | 0.76(0.04) | 0.08(0.05) |  |  |  |  |  | 94.88 |  |
| **HP12** | **gl Si(5)** | 67.63(1.00) | 0.41(0.02) | 9.65(0.29) | 7.21(0.11) | 0.25(0.07) | 0.55(0.02) | 1.30(0.04) | 1.96(0.06) | 4.17(0.02) | 1.09(0.08) | 0.12(0.02) |  | 94.34 | 2.61(0.05) |
|  | **gl Fe(3)** | 22.69(0.62) | 1.58(0.60) | 2.98(0.02) | 37.09(0.40) | 1.78(0.09) | 4.18(0.66) | 6.91(1.40) | 0.79(0.40) | 0.69(0.00) | 18.07(0.98) | 0.23(0.01) |  | 96.99 | 1.59(0.04) |
|  | **Mt(8)** | 0.23(0.14) | 7.15(1.14) | 1.40(0.22) | 84.36(0.88) | 0.67(0.23) | 0.64(0.07) | 0.15(0.08) |  |  |  |  |  | 94.51 |  |
|  | **Fa** | 28.51 | 3.89 | 6.20 | 56.03 | 0.60 | 0.60 | 0.50 |  |  |  |  |  | 96.34 |  |
| **HP13** | **gl Si(4)** | 72.59(0.35) | 0.48(0.09) | 11.54(0.11) | 4.56(0.71) | 0.05(0.03) | 0.05(0.02) | 0.51(0.06) | 2.27(0.46) | 5.16(0.09) | 0.62(0.17) | 0.19(0.01) |  | 98.04 |  |
|  | **Mt(7)** | 0.35(0.17) | 21.44(2.07) | 2.85(0.45) | 71.93(3.13) | 0.44(0.08) | 0.20(0.09) | 0.07(0.07) |  |  |  |  |  | 97.28 |  |
|  | **Fa(5)** | 29.39(0.23) | 0.22(0.09) | 0.05(0.03) | 68.51(0.20) | 1.14(0.10) | 1.80(0.03) | 0.16(0.02) |  |  |  |  |  | 100.82 |  |
| **HP14** | **gl Si(5)** | 73.90(0.32) | 0.26(0.04) | 10.08(0.11) | 5.09(0.11) | 0.14(0.04) | 0.19(0.02) | 0.55(0.06) | 2.35(0.10) | 4.63(0.08) | 0.47(0.03) | 0.13(0.01) |  | 97.79 |  |
|  | **Mt(14)** | 0.37(0.07) | 12.01(1.35) | 2.11(0.14) | 79.56(0.91) | 0.59(0.04) | 0.46(0.04) | 0.06(0.01) |  |  |  |  |  | 95.23 |  |
|  | **Fa(2)** | 29.91(0.49) | 0.17(0.01) | 0.03(0.01) | 58.96(0.74) | 2.03(0.00) | 5.89(0.13) | 0.14(0.04) |  |  |  |  |  | 97.12 |  |
| **HP15** | **gl Si(4)** | 70.69(0.40) | 0.51(0.18) | 11.98(0.25) | 5.84(0.12) | 0.10(0.06) | 0.07(0.03) | 1.00(0.05) | 2.43(0.13) | 5.00(0.12) | 0.68(0.04) | 0.10(0.01) |  | 98.40 |  |
|  | **Mt(8)** | 0.28(0.20) | 25.52(0.75) | 2.78(0.15) | 67.68(2.38) | 0.41(0.07) | 0.21(0.03) | 0.06(0.03) |  |  |  |  |  | 96.90 |  |
|  | **Fa(12)** | 29.12(0.67) | 0.23(0.07) | 0.10(0.17) | 66.40(0.88) | 1.03(0.12) | 1.97(0.17) | 0.26(0.10) |  |  |  |  |  | 99.12 |  |
| **HP16** | **gl(5)** | 73.44(0.41) | 0.35(0.04) | 11.03(0.32) | 3.74(0.15) | 0.16(0.02) | 0.52(0.05) | 0.97(0.01) | 3.13(0.32) | 4.87(0.09) | 0.40(0.09) | 0.11(0.02) |  | 98.71 |  |
|  | **Mt(3)** | 0.21(0.07) | 7.32(0.08) | 1.81(0.27) | 80.82(0.48) | 0.94(0.06) | 1.22(0.12) |  |  |  |  |  |  | 92.35 |  |
| **HP17** | **gl(5)** | 73.10(0.53) | 0.30(0.04) | 11.18(0.11) | 3.90(0.23) | 0.15(0.06) | 0.38(0.05) | 0.97(0.08) | 3.26(0.12) | 4.87(0.15) | 0.20(0.09) | 0.10(0.01) |  | 98.40 |  |
|  | **Mt(3)** | 0.35(0.06) | 5.54(0.20) | 1.90(0.19) | 82.35(0.29) | 0.62(0.04) | 1.10(0.04) |  |  |  |  |  |  | 91.89 |  |
| **HP18** | **gl(5)** | 73.66(0.53) | 0.34(0.04) | 10.90(0.38) | 3.57(0.22) | 0.14(0.02) | 0.36(0.06) | 0.97(0.10) | 3.29(0.10) | 4.75(0.06) | 0.39(0.05) | 0.09(0.01) |  | 98.47 |  |
|  | **Mt(4)** | 0.27(0.20) | 5.68(0.69) | 1.28(0.52) | 82.32(0.69) | 0.89(0.22) | 0.86(0.19) |  |  |  |  |  |  | 91.40 |  |
| **HP22** | **gl Si(6)** | 72.20(0.51) | 0.29(0.03) | 11.23(0.17) | 6.59(0.14) | 0.21(0.06) | 0.36(0.03) | 1.21(0.03) | 2.17(0.06) | 4.67(0.04) | 0.54(0.05) | 0.13(0.01) | 0.06(0.03) | 99.65 |  |
|  | **gl Fe(6)** | 32.21(0.77) | 1.26(0.05) | 4.22(0.13) | 39.13(0.48) | 1.12(0.08) | 2.27(0.09) | 6.43(0.07) | 0.78(0.09) | 0.55(0.03) | 8.55(0.40) | 0.30(0.01) | 1.29(0.03) | 98.11 |  |
|  | **Mt(5)** | 0.35(0.05) | 11.26(0.07) | 2.27(0.05) | 82.61(0.41) | 0.46(0.06) | 0.58(0.02) | 0.08(0.03) |  |  |  |  |  | 97.62 |  |
|  | **Fa(3)** | 30.04(1.11) | 0.12(0.01) | 0.03(0.06) | 60.83(0.67) | 1.47(0.03) | 7.85(0.36) | 0.24(0.03) |  |  |  |  |  | 99.78 |  |
| **HP23** | **gl Si(6)** | 72.12(0.67) | 0.25(0.03) | 11.08(0.22) | 6.58(0.27) | 0.26(0.05) | 0.52(0.04) | 1.11(0.04) | 2.12(0.07) | 4.54(0.02) | 0.60(0.10) | 0.17(0.01) | 0.05(0.03) | 99.39 |  |
|  | **gl Fe(6)** | 28.26(0.98) | 0.91(0.11) | 2.44(0.30) | 40.84(1.05) | 2.73(0.24) | 2.95(0.16) | 6.02(0.22) | 0.57(0.11) | 0.25(0.07) | 10.66(0.45) | 0.38(0.02) | 1.22(0.09) | 97.25 |  |
|  | **Mt(5)** | 0.33(0.06) | 7.21(0.06) | 1.62(0.09) | 86.27(0.48) | 0.67(0.05) | 0.57(0.03) | 0.03(0.01) |  |  |  |  |  | 96.73 |  |
|  | **Fa(5)** | 31.17(0.45) | 0.16(0.02) | 0.04(0.03) | 56.22(0.49) | 2.46(0.05) | 10.09(0.81) | 0.18(0.05) |  |  |  |  |  | 100.32 |  |
| **HP24** | **gl Si(6)** | 72.86(0.54) | 0.31(0.02) | 11.31(0.21) | 5.93(0.09) | 0.20(0.06) | 0.44(0.03) | 1.17(0.04) | 2.17(0.09) | 4.63(0.04) | 0.66(0.07) | 0.10(0.01) | 0.03(0.02) | 99.81 |  |
|  | **gl Fe(6)** | 26.28(0.37) | 1.21(0.09) | 3.68(0.36) | 36.12(0.49) | 1.49(0.07) | 3.53(0.04) | 8.40(0.14) | 0.63(0.08) | 0.41(0.03) | 14.71(0.23) | 0.25(0.01) | 1.03(0.04) | 97.74 |  |
|  | **Mt(5)** | 0.28(0.07) | 9.15(0.06) | 2.39(0.08) | 82.76(0.78) | 0.48(0.04) | 0.68(0.03) | 0.04(0.02) |  |  |  |  |  | 95.78 |  |
| **HP25** | **gl Si(6)** | 72.48(0.71) | 0.38(0.03) | 11.13(0.58) | 5.70(0.46) | 0.13(0.04) | 0.20(0.04) | 1.05(0.10) | 2.48(0.12) | 4.99(0.06) | 0.44(0.06) | 0.15(0.02) | 0.05(0.02) | 99.14 |  |
|  | **gl Fe(4)** | 29.15(1.16) | 2.82(0.29) | 5.53(0.21) | 37.96(0.89) | 0.99(0.13) | 1.48(0.30) | 6.38(0.38) | 0.57(0.07) | 0.43(0.04) | 10.98(0.69) | 0.41(0.17) | 1.34(0.10) | 96.70 |  |
|  | **Mt(5)** | 0.82(0.76) | 22.25(0.23) | 3.21(0.38) | 71.90(0.55) | 0.55(0.08) | 0.46(0.03) | 0.06(0.03) |  |  |  |  |  | 99.24 |  |
|  | **Fa(5)** | 30.67(0.54) | 0.09(0.06) | 0.10(0.07) | 63.03(1.66) | 1.32(0.07) | 5.34(1.01) | 0.24(0.02) |  |  |  |  |  | 100.79 |  |
| **HP26** | **gl Si(6)** | 72.89(0.35) | 0.20(0.02) | 11.10(0.10) | 5.92(0.11) | 0.20(0.05) | 0.35(0.03) | 1.00(0.06) | 2.20(0.09) | 4.55(0.06) | 0.58(0.07) | 0.11(0.01) | 0.04(0.02) | 99.14 |  |
|  | **gl Fe(4)** | 29.31(0.17) | 0.81(0.04) | 4.06(0.30) | 37.76(0.49) | 1.74(0.17) | 3.27(0.15) | 6.43(0.14) | 0.67(0.05) | 0.47(0.04) | 11.76(0.35) | 0.33(0.13) | 1.07(0.03) | 97.68 |  |
|  | **Mt(10)** | 0.42(0.11) | 7.50(0.15) | 1.70(0.11) | 85.16(0.63) | 0.62(0.08) | 0.47(0.03) | 0.06(0.07) |  |  |  |  |  | 95.93 |  |
|  | **Fa(5)** | 30.66(0.76) | 0.16(0.05) | 0.06(0.06) | 58.50(0.61) | 1.82(0.09) | 8.77(0.21) | 0.19(0.02) |  |  |  |  |  | 100.16 |  |
| **HP27** | **gl Si(7)** | 72.63(0.66) | 0.27(0.02) | 11.36(0.25) | 5.81(0.28) | 0.17(0.07) | 0.30(0.05) | 1.10(0.06) | 2.25(0.09) | 4.72(0.07) | 0.52(0.06) | 0.10(0.01) | 0.04(0.01) | 99.27 |  |
|  | **gl Fe(7)** | 29.03(0.67) | 1.09(0.10) | 4.05(0.13) | 38.01(0.40) | 1.27(0.08) | 2.59(0.16) | 7.33(0.37) | 0.77(0.07) | 0.45(0.02) | 11.72(0.64) | 0.25(0.02) | 1.53(0.06) | 98.09 |  |
|  | **Mt(5)** | 0.36(0.03) | 9.19(0.32) | 1.83(0.08) | 83.55(1.32) | 0.53(0.05) | 0.36(0.04) | 0.05(0.02) |  |  |  |  |  | 95.88 |  |
|  | **Fa(5)** | 30.57(0.33) | 0.10(0.04) | 0.07(0.03) | 62.33(0.43) | 1.77(0.14) | 5.78(0.15) | 0.23(0.01) |  |  |  |  |  | 100.85 |  |

^1^Number of microprobe analyses;

^2^One standard deviation of replicate analyses is reported in parentheses

Abbreviation: gl Si=Si-rich glass, gl Fe=Fe-rich glass. Other abbreviations are same as in Supplementary Table 3.
